# DHX8 Plays a Critical Role in Larval Development in Lepidopteran *Bombyx mori*

**DOI:** 10.3390/insects17030236

**Published:** 2026-02-25

**Authors:** Ling Ding, Cexin Xu, Yunxiao Zhang, Yuanbo Wang, Yong Hou, Guanwang Shen, Ping Lin, Qingyou Xia, Ping Zhao, Zhiqing Li

**Affiliations:** 1 Integrative Science Center of Germplasm Creation in Western China (CHONGQING) Science City, Chongqing Technology Innovation Center of Breeding, Biological Science Research Center, Southwest University, Chongqing 400715, China; m13290042003@163.com (L.D.); 18811763797@163.com (C.X.); 15719425550@139.com (Y.Z.); wyb1707@163.com (Y.W.); yhou@swu.edu.cn (Y.H.); gwshen@swu.edu.cn (G.S.); linpingswu@swu.edu.cn (P.L.); xiaqy@swu.edu.cn (Q.X.); zhaop@swu.edu.cn (P.Z.); 2 Key Laboratory for Germplasm Creation in Upper Reaches of the Yangtze River, Ministry of Agriculture and Rural Affairs, Chongqing 400715, China

**Keywords:** silkworm, CRISPR-Cas9, DHX8, RNA splicing

## Abstract

DHX8 encodes a DEAH-box RNA helicase, an ATP-dependent enzyme that plays essential roles in RNA metabolism, including pre-mRNA splicing, transcription, and mRNA decay. Although DHX8 dysfunction has been linked with developmental abnormalities and disease pathogenesis in multiple model organisms, its biological functions in Lepidoptera, particularly in the silkworm *Bombyx mori*, remain unknown. To investigate the developmental role of *B*. *mori* DHX8 (BmDHX8), we generated knockout mutants using CRISPR-Cas9 genome editing. Genome sequencing confirmed frameshift mutations in the BmDHX8 locus. BmDHX8 mutants exhibited severe developmental defects such as dramatically reduced body size and premature lethality of silkworm larvae. Molecular characterization suggested systemic dysregulation, as evidenced by decreased triglyceride accumulation, impaired mTOR signaling activity, and increased aberrant splicing events. Therefore, these results indicate that loss of BmDHX8 is associated with aberrant splicing and alterations in lipid homeostasis and mTOR signaling pathways, potentially contributing to developmental defects. Taken together, our study offers an initial functional knockout analysis of BmDHX8 in regulating larval development in silkworms.

## Simple Summary

The spliceosome, an essential macromolecular complex in eukaryotic cells, catalyzes both constitutive and alternative splicing of intron-containing pre-mRNAs to generate mature transcripts for protein synthesis. As a key component of the spliceosome, DEAH-box helicase 8 (DHX8) is essential for efficient splicing of the pre-mRNA and thereby underpins multiple biological processes. In this study, we explored the role of *Bombyx mori* DHX8 (BmDHX8) in silkworm development through CRISPR-Cas9-mediated knockout. Disruption of BmDHX8 led to severe developmental defects, including dwarfism and early death of silkworm larvae. Further study suggested these phenotypes were associated with RNA splicing errors that altered lipid and nutrient signaling readouts. Our findings thus indicate that BmDHX8 functions as a regulator linking RNA processing to nutrient homeostasis during silkworm larval development.

## 1. Introduction

In eukaryotes, gene expression requires the removal of non-coding introns through pre-mRNA splicing [1]. While this process is normally tightly controlled, aberrant splicing is increasingly linked to human diseases, especially cancer [2,3,4]. Splicing is catalyzed by the spliceosome, a dynamic ribonucleoprotein complex consisting of five small nuclear ribonucleoproteins (snRNPs) and approximately 200 auxiliary proteins [5]. Following splicing, the spliceosome releases mature mRNA via conformational and compositional changes, mediated by at least eight helicase superfamily 2 (SF2) RNA helicases dependent on nucleotide triphosphate (NTP) [6,7,8]. Based on variations in sequence motifs, structural features, and mechanisms, the SF2 helicase family is further divided into three subfamilies including DEAD-box, DEAH-box, and Ski2-like [9,10].

DEAH-box helicase 8 (DHX8) belongs to the DEAH-box subfamily. It is an essential RNA helicase that mediates mature mRNA release from the spliceosome [11,12]. Structural analysis reveals multiple functional domains in DHX8, including an RNA-binding domain (S1), DEAH/DEAD box, C-terminal helicase domain, and HA2 domain, which collectively confer ATP binding, RNA binding, and RNA helicase activities [13,14]. Functional studies demonstrate that RNA silencing of DHX8 in human Hela cells disrupts cell division, while mutation in zebrafish impairs splicing of hematopoietic-related mRNAs [15,16]. The yeast homolog Prp22 similarly facilitates mRNA dissociation from U5 snRNP in post-splicing complexes and functions in splicing fidelity control [13,17]. These findings highlight dual roles of DHX8 in both mRNA splicing and cell cycle regulation. Despite these advances, the biological function of DHX8 in insects remains poorly characterized. Recent isolation of the Drosophila spliceosomal gene Prp22 underlines its critical role in nurse-cell chromatin dispersal [18].

The silkworm *Bombyx mori* serves as an important lepidopteran model insect with distinct growth and developmental phases [19]. During the larval growth period, silkworms actively assimilate nutrients to support rapid somatic growth and organogenesis, establishing the foundation for subsequent development. The transition involves four molting cycles with significant physiological changes, culminating in metamorphosis from pupa to adult [19]. The growth and development of silkworms are precisely regulated by the juvenile hormone (JH) and molting hormone (MH), with their titers critically determining molting progression and final body size [20,21,22]. Lipids, as essential metabolic substrates, play vital roles in energy storage, hormone synthesis, and signal transduction [23,24,25]. In silkworm, lipid metabolism is modulated through the integration of environmental cues and internal signals, with nutritional factors and endocrine hormones being key regulators of lipid homeostasis [26]. As an economically vital species, understanding its growth patterns also holds significant implications for sericulture applications.

In this study, we utilized the CRISPR-Cas9 (Clustered Regularly Interspaced Short Palindromic Repeats/CRISPR-associated protein 9) system [27,28] to generate targeted mutations in the *BmDHX8* gene of *B*. *mori*. We successfully generated a BmDHX8 knockout silkworm model, which exhibited severe developmental defects such as growth retardation, lower survival rate, and a 40% reduction in body weight compared to wild-type silkworms. Further investigations revealed systemic metabolic disturbances of impaired nutrient utilization and lipid homeostasis, as well as defective splicing of mTOR pathway-related genes. These findings collectively demonstrate that the RNA helicase BmDHX8, functioning as a key splicing regulator, plays a critical role in silkworm growth and development.

## 2. Materials and Methods

### 2.1. Cell Line

The BmE cell line was derived from silkworm embryos [29,30], and cultured in Grace medium (Gibco, Waltham, MA, USA) supplemented with 10% fetal bovine serum (FBS, Hyclone, Logan, UT, USA) and penicillin–streptomycin (Thermo Fisher Scientific, Waltham, MA, USA) at 27 °C.

### 2.2. Silkworm Strains

The silkworm strains used in this experiment are Dazao and Nistari-Nos-Cas9, which were maintained in our laboratory [31]. The silkworm eggs are kept under conditions at a temperature of 27 °C and a relative humidity of 65–80%, and larvae are fed with fresh mulberry leaves at room temperature (25 °C).

### 2.3. Sequence Analysis

From NCBI, we downloaded the annotated DHX8 protein sequences, including *Bombyx mori* (XP_004921601), *Homo sapiens* (NP_004932), *Mus musculus* (NP_659080), *Danio rerio* (XP_021336018), *Drosophila melanogaster* (NP_610928), *Manduca sexta* (XP_030023536), *Aedes aegypti* (XP_021697878), *Anopheles gambiae* (XP_308573), *Danaus plexippus* (XP_061383398), *Tribolium castaneum* (XP_008198238), *Apis mellifera* (XP_623289), *Spodoptera litura* (XP_022819323), and *Saccharomyces cerevisiae* (CAA41530). Multiple sequence alignment of the functional domain sequences was performed. A phylogenetic tree was then constructed using the neighbor-joining with 1000 bootstrap replicates in CLC sequence viewer 7 (https://clc-sequence-viewer.software.informer.com/, accessed on 26 September 2024), a method chosen for its computational efficiency in the initial screening of our dataset.

### 2.4. Plasmid Construction

For the expression of *BmDHX8*, its full-length cDNA was amplified from the cDNA library of cultured silkworm cells by using primers listed in Table S1, and further cloned into a pENTR11 (Invitrogen, Carlsbad, CA, USA) vector as described previously [30]. The pENTR11 clone of BmDHX8 gene was inserted into the expression vector of pPBO_ie2GW (containing N-terminal EGFP tag) via LR recombination reaction to construct the EGFP-DHX8 expression vector. All plasmids were sequenced to verify the clones.

For the construction of DHX8-gRNA, the CCTop CRISPR-Cas9 target online predictor (https://cctop.cos.uni-heidelberg.de:8043/, accessed on 30 March 2022) [32] was used to design gRNA for *BmDHX8* gene. Table S1 lists all the gRNA and primer sequences. The plasmid piggyBac[EGFP,DHX8-gRNA] was constructed to express DHX8-gRNA under the control of U6 promoter and the *EGFP* fluorescence gene was used as a selective marker.

### 2.5. Generation of BmDHX8 Mutant Silkworm

The transgenic silkworm expressing gRNA targeting *BmDHX8* was generated using piggyBac-mediated germline transformation, following previously reported methods [30]. To initiate somatic mutagenesis, the Nistari-Nos-Cas9 strain (constitutively expressing Cas9 protein) was crossed with the BmDHX8 gRNA transgenic strain. F1 progeny exhibiting dual fluorescence (indicating inheritance of both the Cas9 and DHX8-gRNA transgenes) were selected as potential BmDHX8 mutants. Successful genomic editing at the *BmDHX8* locus in these individuals was confirmed by PCR amplification. We established a *BmDHX8* mutant strain by selecting individuals with PCR-verified mutations including both homozygotes and heterozygotes. This strain was subsequently used for phenotypic and molecular analyses.

### 2.6. Genomic DNA Extraction and Mutagenesis Analysis

Genomic DNA was extracted from the molted epidermis during larvae to pupae for mutation analysis. To confirm CRISPR-Cas9-induced mutations in the *BmDHX8* gene, the genomic region encompassing the gRNA target site was amplified by PCR using primers listed in Table S1. To resolve potential mosaicism and characterize individual mutant alleles, the PCR products were cloned into the pEASY^®^-Blunt Zero Cloning Vector (TransGen Biotech, Beijing, China). Multiple independent clones (typically 8–10 per individual) were picked and subjected to deep sequencing of amplicons (BGI, Shenzhen, China). The resulting sequences were analyzed to identify insertion/deletion mutations at the target site and the related individuals were crossed to establish a *BmDHX8* mutant strain.

### 2.7. Spatio-Temporal Expression Analysis

To investigate the spatio-temporal expression profile of *BmDHX8*, samples were prepared from silkworm tissues dissected at day 3 of the fifth instar larvae and different developmental stages including larvae, pupae, and moths. All these samples were stored in liquid nitrogen for subsequent real-time quantitative PCR (RT-qPCR) analysis.

### 2.8. Real-Time Quantitative PCR Analysis

Total RNA from different samples was separately extracted using Total RNA Kit (Omega, Biel/Bienne, Switzerland), and two μg of which were reverse-transcribed by the GoScript Reverse Transcription System (Promega, Madison, WI, USA) to obtain corresponding cDNA. The same amount of cDNA was used for the RT-qPCR assay using an SYBR Premix Ex TaqTM II kit (TaKaRa & Clontech, Dalian, China) on the Applied Biosystems 7500 Fast Real-Time PCR System (Applied Biosystems, Foster City, CA, USA). *Eukaryotic translation initiation factor 4A* (*eIF-4a*) was selected as the reference gene [31]. Each experiment was independently repeated three times, and relative mRNA levels were determined using the 2^−ΔΔCt^ method [33]. All primers for RT-qPCR are listed in Table S1.

### 2.9. Subcellular Localization

Plasmids encoding EGFP-DHX8 or EGFP were transfected into BmE cells. Forty-eight hours after transfection, cells were washed once with PBS and fixed with 4% paraformaldehyde in PBS for 10 min. After permeabilization with 0.1% Triton X-100 for 10 min, the nuclei DNA was counterstained by 4′,6-diamidino-2-phenylindole (DAPI) (Invitrogen, Carlsbad, CA, USA). Fluorescence imaging was captured by a fluorescent microscope (Leica, Wetzlar, Germany).

### 2.10. Phenotypic Observation

During our rearing of DHX8-KO mutant silkworms, significant developmental delays were observed compared to wild-type (WT) controls. To record the mutant phenotypes, the photos of larvae were captured across different stages. Larval body weight was monitored in both WT and BmDHX8 mutant strains from the second instar to the wandering phase, with 15 individuals sampled per genotype at each corresponding developmental stage. Survival ratio was also calculated as the percentage of larvae successfully spinning cocoons.

### 2.11. Oil Red O Staining

Lipid accumulation in fat body was analyzed by whole-mount Oil Red O histochemistry coupled with spectrophotometric triglyceride measurement. Freshly dissected fat body tissues from DHX8-KO and WT controls were fixed in 4% paraformaldehyde for 1 h at room temperature. After PBS rinsing, tissues were stained with 0.5% Oil Red O working solution (Beyotime, Shanghai, China) for 10 min with gentle agitation. Following three PBS washes, whole-mount specimens were mounted in 80% glycerol/PBS and imaged immediately under an inverted microscope.

For triglyceride (TG) quantification, fat body samples (5 individuals per group) were homogenized in chloroform–methanol (2:1 *v*/*v*) at 10% (*w*/*v*). Homogenates were centrifuged at 12,000× *g* (20 min, 4 °C). The organic phase was collected and evaporated under nitrogen. Triglyceride content was determined and normalized to protein content using the Triglyceride Assay Kit (Beyotime, Shanghai, China) with glycerol standards under spectrophotometric absorbance at 520 nm. Three independent biological replicates were performed.

### 2.12. RNA Splicing Analysis

Genomic DNA and RNA samples were isolated from the fat body of DHX8-KO and WT. We selected genes whose expression was altered in *DHX8* knockout mutants and performed PCR using primers targeting adjacent exons spaced less than 1000 bp apart [16]. Among these, we focused on genes involved in lipid metabolism and the mTOR pathway, but only a limited set, including *TOR1* and *4EBP*, met this criterion for further splicing analysis (Table S1).

### 2.13. Statistical Analysis

All data were expressed as the mean ± standard deviation (SD) of three independent biological replicates. Statistical significance was analyzed using Student’s t test when compared DHX8-KO to WT for each experiment, and expressed as follows: * *p* < 0.05, ** *p* <0.01, and *** *p* < 0.001.

## 3. Results

### 3.1. RNA Helicase DHX8 Is Highly Conserved in Different Species

To investigate the function of RNA helicase DHX8 in silkworm, we first identified DHX8 in the silkworm genome by using human DHX8 protein sequence as inquiry through the BLASTP program (https://blast.ncbi.nlm.nih.gov/Blast.cgi, accessed on 20 December 2021) in the NCBI. As a result, an annotated *BmDHX8* (NCBI accession number: XP_004921601) in silkworm was obtained. We further cloned the full-length *BmDHX8* from the cDNA library of silkworm cells (Figure S1). After sequencing, it was 3945 bp in length, which was consistent with the annotated *BmDHX8* gene.

**Figure 1:**
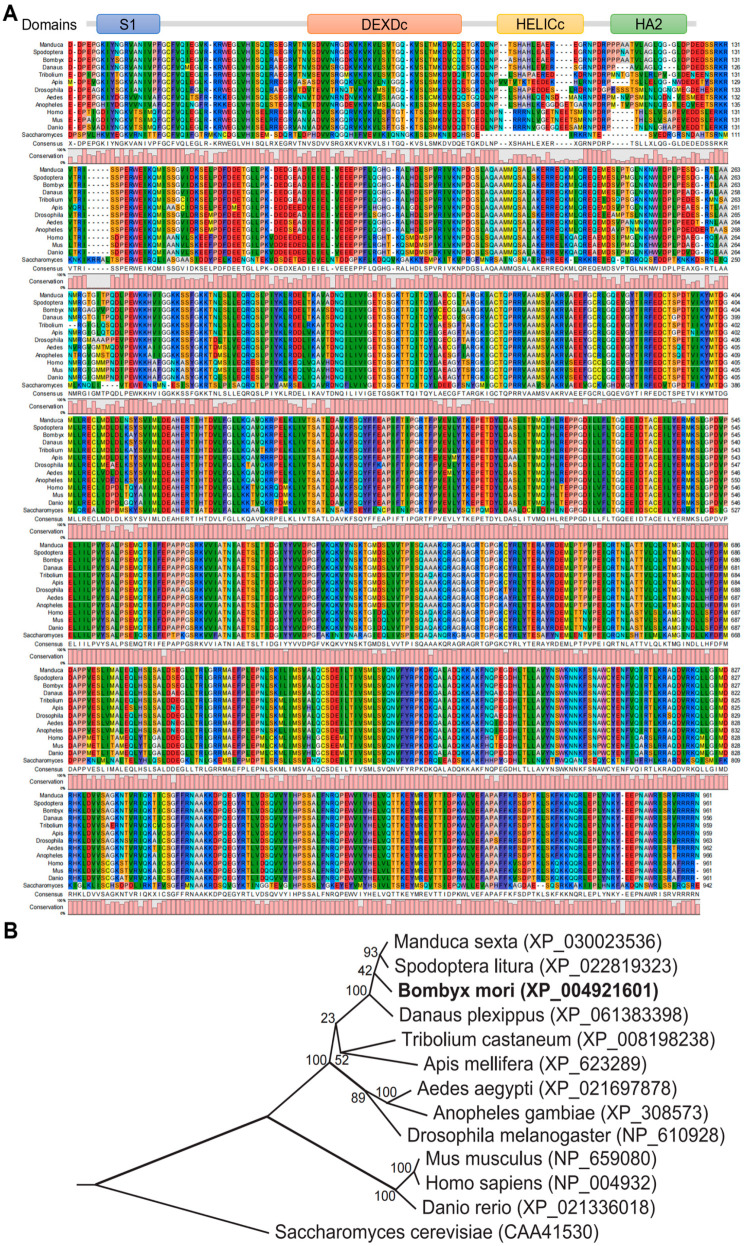
Identification of BmDHX8 in silkworm. (**A**) Multiple sequence alignment of DHX8 functional domains from different species. Schematic diagram of DHX8 functional domains is shown and used for multiple sequence alignment. (**B**) Phylogenetic analysis of DHX8 from different species. The highlight (bold font) indicates the DHX8 protein sequence from *Bombyx mori*.

Structural analysis of silkworm BmDHX8 showed that it contains four typical domains of S1, DEXDc, HELICc, and HA2 (Figure 1A). We compared protein sequences containing these four domains from different species including mammals, insects, and yeast. It was interestingly shown that they are very conserved in distinct species (Figure 1A). Based on multiple sequence alignments, we also constructed the phylogenetic tree of DHX8 using the neighbor-joining method. As shown in Figure 1B, DHX8 from lepidopteran insects was clustered together, and separated from other insects. All insect DHX8 formed a different clade with mammals. Therefore, our identification and analysis of DHX8 reveals it to be a highly conserved RNA helicase, underscoring its functional constraint and evolutionary preservation across diverse species.

### 3.2. BmDHX8 Is Widely Expressed Throughout the Development of Silkworm

**Figure 2:**
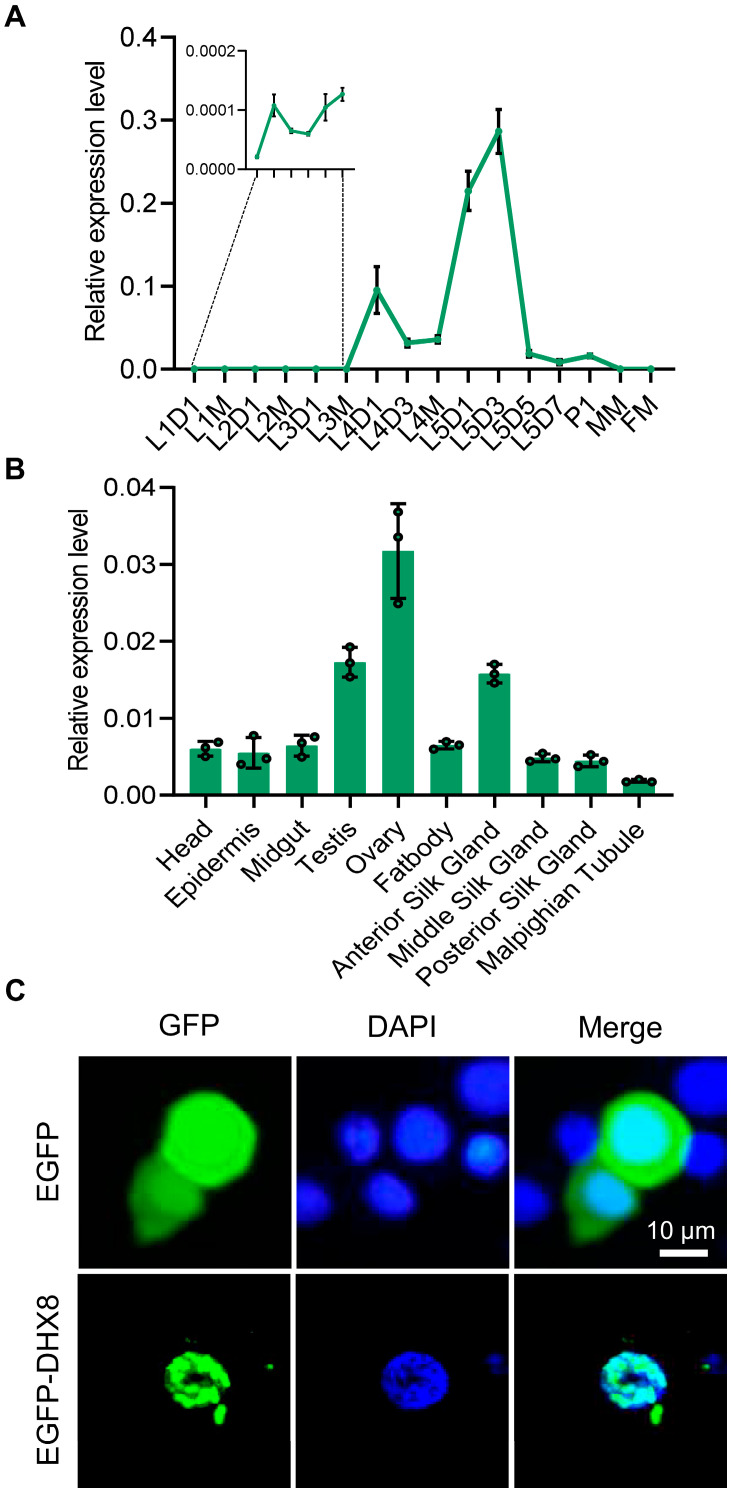
Expression of *BmDHX8* in silkworm. (**A**) Temporal expression profile of *BmDHX8* gene during the different developmental stages. L1D1: day 1 of the first instar larvae; L1M: molting of the first instar larvae; L2D1: day 1 of the second instar larvae; L2M: molting of the second instar larvae; L3D1: day 1 of the third instar larvae; L3M: molting of the third instar larvae; L4D1: day 1 of the forth instar larvae; L4D3: day 3 of the forth instar larvae; L4M: molting of the forth instar larvae; L5D1: day 1 of the fifth instar larvae; L5D3: day 3 of the fifth instar larvae; L5D5: day 5 of the fifth instar larvae; L5D7: day 7 of the fifth instar larvae; P1: day 1 of the pupae; MM: male moth; FM: female moth. (**B**) Spatial expression profile of *BmDHX8* gene in various tissues on day 3 of the fifth instar larvae. (**C**) Subcellular localization of BmDHX8 protein fused with EGFP in BmE cells. Plasmids of pPBO-EGFP or pPBO-EGFP-DHX8 were transfected and transiently expressed in BmE cells. Cell nuclei were stained using DAPI. Scale bar is 10 μm. Data are presented as mean ± SD (n = 3).

We next analyzed the expression profiles of *BmDHX8* in silkworm using RT-qPCR assay. We detected the temporal pattern of *BmDHX8* expression during the different developmental stages, including the larval stage, the pupal stage, and the moth stage. It was shown that the *BmDHX8* gene was expressed in all stages with relatively higher expression in the fifth instar larvae (Figure 2A). Due to the high expression of *BmDHX8* on day 3 of the fifth instar larvae, we then isolated 10 tissues on this stage to analyze its spatial expression pattern. The result showed that *BmDHX8* was also expressed in all tissues and the silkworm gonads had relatively higher levels (Figure 2B).

We also observed the subcellular localization of *BmDHX8* in cultured silkworm BmE cells by using an enhanced green fluorescent protein (EGFP)-fusion expression system. It was shown that the control of EGFP was localized in both cytoplasm and nucleolus, while the fusion expression of EGFP with BmDHX8 was specifically localized in the nucleolus and exhibited dot signals (Figure 2C), which is consistent with a role of DHX8 in RNA processing. All these results imply that BmDHX8 plays critical roles during the whole life cycle of silkworm.

### 3.3. CRISPR-Cas9 System Induces the Mutagenesis of BmDHX8

**Figure 3:**
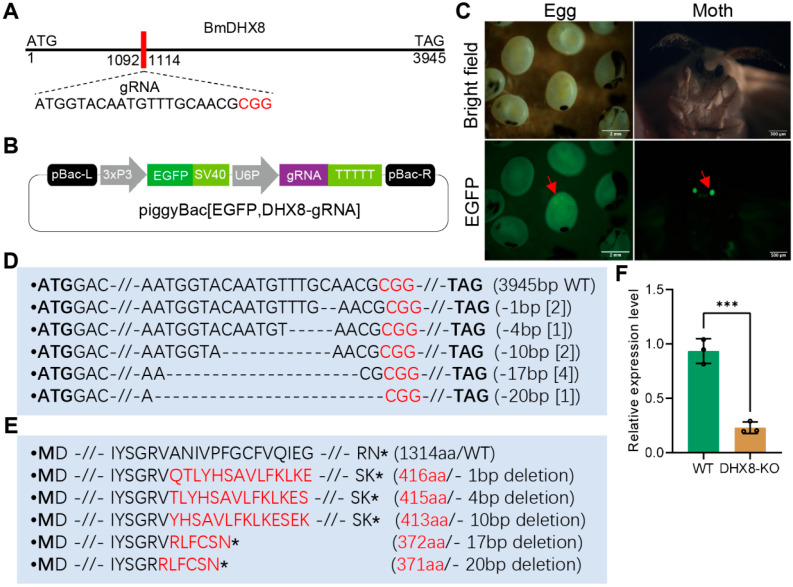
CRISPR-Cas9-mediated mutation of BmDHX8. (**A**) Schematic diagram of *BmDHX8* gene structure and gRNA target site. (**B**) Plasmid construction for the generation of transgenic silkworms. Expression of DHX8-gRNA was controlled by the U6 promoter and the silkworms were selected by EGFP marker. (**C**) After hybridization of DHX8-gRNA and Cas9 transgenic silkworms, the positive silkworm egg and moth were screened under the EGFP fluorescence (green). Scale bars are 2 mm and 500 μm. (**D**) PCR-based amplification and sequencing of regions targeted by gRNA in DHX8-KO and WT silkworms. Various mutations were detected in DHX8-KO silkworms and the WT sequence is shown at the top. The red nucleotides represented the PAM sequence, and the bold nucleotides were start and stop codons. Deletions were indicated by dashed lines. The number within the “[]” represented the number of mutations recovered by deep sequencing of amplicons. (**E**) Mutation on amino acids in DHX8-KO. Amino acids labeled in red were mutations and the asterisks were stop codon. (**F**) The relative mRNA expression of *BmDHX8* in WT and DHX8-KO silkworms was determined by RT-qPCR. Data are presented as mean ± SD (n = 3). For the significant analysis: *** *p* < 0.001.

To study the physiological role of BmDHX8 in silkworm individuals, we established a transgenic CRISPR-Cas9 system to generate the BmDHX8 mutant, as previously described [30,34]. One specific target gRNA sequence against *BmDHX8* was obtained by CCTOP website analysis (Figure 3A). The gRNA of BmDHX8 was annealed and ligated with a gRNA expression vector under the control of the silkworm U6 promoter with the EGFP selective marker (Figure 3B). After obtaining the DHX8-gRNA transgenic strain, they were hybridized with the Cas9 transgenic silkworms, and the positive silkworm egg and moth were screened under the EGFP fluorescence (Figure 3C). We dissected BmDHX8 knockout (DHX8-KO) individuals, extracted their genome, and detected whether *BmDHX8* was successfully knocked out using genomic PCR and sequencing. As shown in Figure 3D,E and Figure S2, *BmDHX8* in the genomes of DHX8-KO mutants had various deletions, which led to the complete loss of the C-terminal functional domains. RT-qPCR confirmed a significant down-regulation of *BmDHX8* expression in DHX8-KO mutants (Figure 3F). The residual expression of *BmDHX8* in the mutant population is likely attributable to the presence of both homozygous and heterozygous individuals. Nevertheless, these results demonstrate that the transgenic CRISPR-Cas9 system can effectively knock out the *BmDHX8* gene in silkworm.

### 3.4. Knockout of BmDHX8 Results in Development Defects of Silkworm

**Figure 4:**
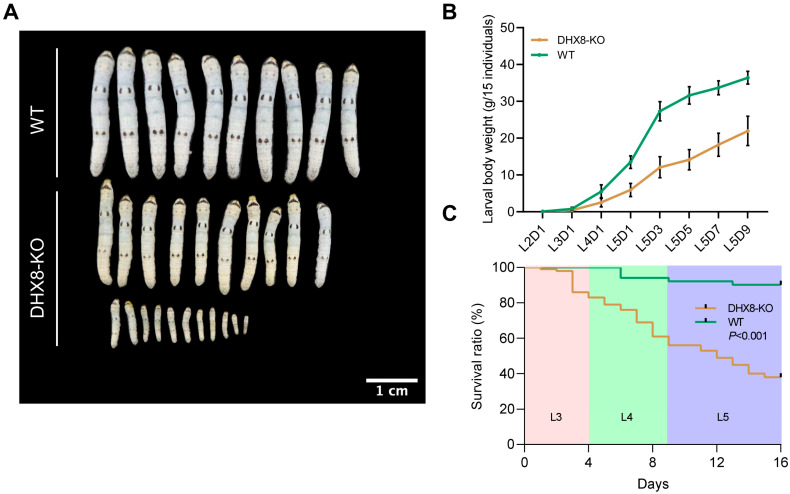
Knockout of *BmDHX8* induced development defects of silkworm. (**A**) BmDHX8 mutation caused a significant decrease in body size of the fifth instar larvae. Scale bar is 1 cm. (**B**) Body weight analysis between DHX8-KO and WT silkworms at each corresponding developmental larval stage. Data are presented as mean ± SD (n = 3). (**C**) Survival ratio analysis of DHX8-KO and WT silkworms during the third (L3), forth (L4), and fifth (L5) instar larvae.

During our rearing of DHX8-KO mutant silkworms, we found that when wild-type (WT) individuals were developed to the fifth instar larvae, a large number of DHX8-KO mutants were still at the third instar larvae, and the larval development of DHX8-KO mutants was remarkably delayed. Moreover, most mutants exhibited a markedly reduced body size across the larval stage (Figure 4A). We measured the larval body weight and found that the DHX8-KO mutants were significantly lighter than the WT at each corresponding developmental stage, showing a 40% reduction on the final larval day (Figure 4B). We also assessed the survival of DHX8-KO mutants and WT individuals from larval hatching. The results revealed that only 38% of the mutants survived to the pupal stage (Figure 4C). The above observations imply that BmDHX8 is required for normal growth and development of silkworm larvae.

### 3.5. Knockout of BmDHX8 Disrupts Lipid Accumulation in the Fat Body of Silkworm

**Figure 5:**
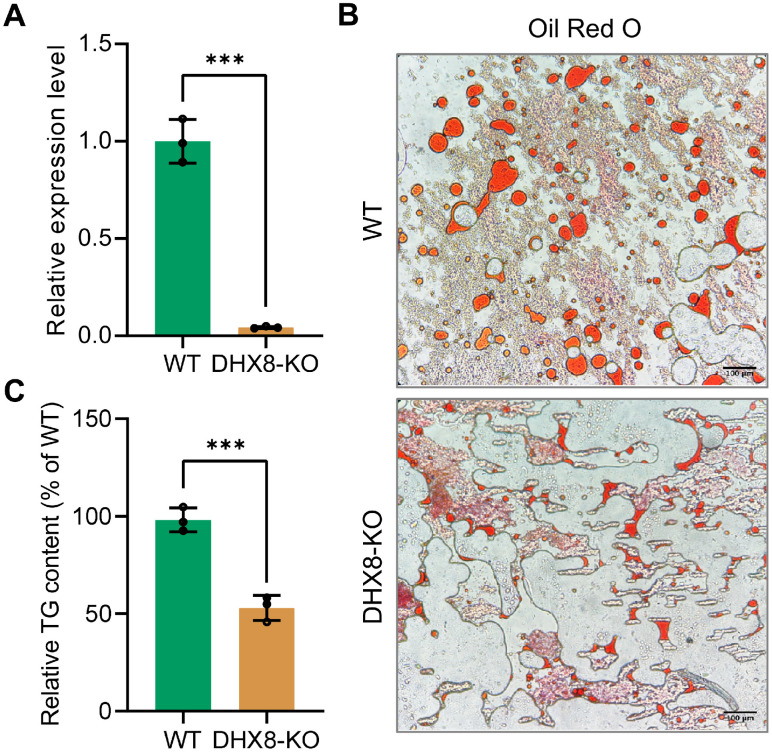
Knockout of *BmDHX8* disrupted lipid accumulation in the fat body of silkworm. (**A**) Relative expression of *BmDHX8* was analyzed by RT-qPCR using the fat body from WT and DHX8-KO silkworms on day 3 of the fifth instar larvae. (**B**) Oil red O staining showed a reduction of lipid droplets in the fat body of DHX8-KO compared to WT. (**C**) Relative TG content in the fat body of WT and DHX8-KO silkworms. Data are presented as mean ± SD (n = 3). For the significant analysis: *** *p* < 0.001.

The reduced body size of silkworms following BmDHX8 knockout, coupled with the importance of lipid absorption, storage, and utilization as key energy sources for organismal growth and development, led us to investigate whether BmDHX8 is involved in silkworm energy metabolism. We therefore focused on the fat body, examining lipid storage measurements on triglyceride (TG), which is the major form of stored fat in the insect fat body [24,25]. We further confirmed the expression level of *BmDHX8* in the silkworm fat body on day 3 of the fifth instar larvae after BmDHX8 knockout. It was shown that *BmDHX8* expression was significantly down-regulated in the DHX8-KO fat body (Figure 5A).

In silkworm, lipid droplets including TG are highly accumulated as nutrient storage in the fat body of the final larval stage, which will be metabolized to provide energy for pupae and later moths. To determine whether BmDHX8 plays a role in regulating lipid accumulation, we examined the lipid droplets in the fat body on day 3 of the fifth instar larvae using Oil Red O staining. We found that knockout of *BmDHX8* decreased lipid droplets in the fat body compared to the WT silkworms (Figure 5B). Subsequently, we quantified TG contents using the Triglyceride Assay Kit and found that about half of TG contents were reduced in BmDHX8 mutants (Figure 5C).

### 3.6. Knockout of BmDHX8 Disordered the Lipid Metabolism

**Figure 6:**
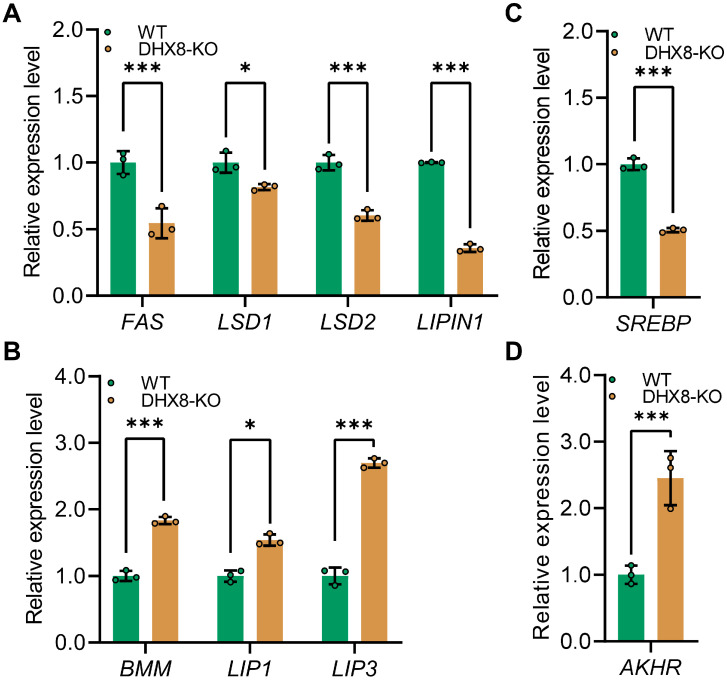
Knockout of *BmDHX8* disordered the expression of lipid metabolism genes in the fat body of silkworm. (**A**) Relative expression of lipid synthesis-related genes in DHX8-KO and WT silkworms. (**B**) Relative expression of lipid mobilization-related genes in DHX8-KO and WT silkworms. (**C**) Relative expression of *SREBP* in DHX8-KO and WT silkworms. (**D**) Relative expression of *AKHR* in DHX8-KO and WT silkworms. Data are presented as mean ± SD (n = 3). For the significant analysis: * *p* < 0.05, *** *p* < 0.001.

To understand the relevance of BmDHX8 function in lipid accumulation, we next asked whether impaired lipid accumulation due to knockout of *BmDHX8* would affect lipid synthesis (lipogenesis) and lipid mobilization (lipolysis). For this, we analyzed lipogenesis-related genes including *fatty acid synthetase* (*FAS*), *lipid storage droplet* (*LSD1* and *LSD2*), and *LIPIN1*, as well as lipolysis-related genes such as *brummer* (*BMM*), and *lipases* (*LIP1* and *LIP3*) [35,36,37]. It was shown that *BmDHX8* depletion significantly decreased the expression of lipogenesis-related genes (Figure 6A), and increased the expression of lipolysis-related genes (Figure 6B). We also detected the expression of *sterol regulatory element-binding proteins* (*SREBP*), a transcription factor that is involved in the biosynthesis of fatty acids [38], and *adipokinetic hormone receptor* (*AKHR*), an adipokinetic hormone pathway that initiates the lipolysis of storage fat [39]. Consistent with the expression of lipid metabolism genes, *SREBP* was down-regulated (Figure 6C), whereas *AKHR* was significantly upregulated (Figure 6D). These findings indicate that BmDHX8 regulates the transcriptional expression of genes involved in both adipogenesis and lipolysis, and its depletion may disrupt lipid metabolism, thereby causing developmental defects in silkworm.

### 3.7. Knockout of BmDHX8 Inhibits the mTOR Pathway

**Figure 7:**
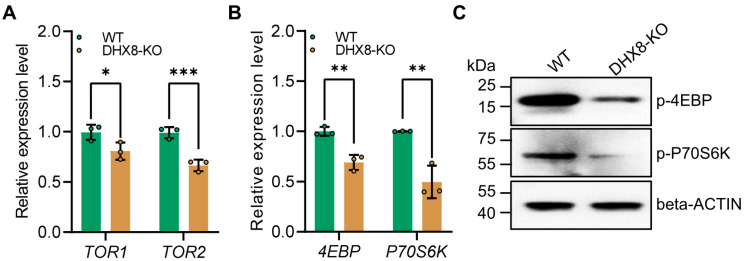
Knockout of *BmDHX8* inhibited the expression of mTOR signaling genes in the fat body of silkworm. (**A**) Relative expression of the mTOR signaling genes in the fat body of WT and DHX8-KO silkworms. (**B**) Relative expression of mTOR downstream genes in the fat body of WT and DHX8-KO silkworms. (**C**) Phosphorylation levels of mTOR downstream genes in the fat body of WT and DHX8-KO silkworms were detected by using p-4EBP and p-P70S6K antibodies, and beta-ACTIN was used as a loading control. Data are presented as mean ± SD (n = 3). For the significant analysis: * *p* < 0.05, ** *p* < 0.01, *** *p* < 0.001.

The mammalian target of the rapamycin (mTOR) signaling pathway is crucial for regulating various physiological processes such as cell growth, proliferation, and metabolism [40,41]. Previous studies showed that mutations in the TOR gene can also cause Drosophila fat bodies to exhibit features of decreased lipid levels [35]. Therefore, we examined the expression of key genes including *TOR1* and *TOR2* in the mTOR pathway using RT-qPCR. It was shown that in the DHX8-KO fat body, *TOR1* and *TOR2* were significantly down-regulated (Figure 7A), indicating that knockout of *BmDHX8* inhibited the mTOR signaling pathway. Recent studies revealed that the mTOR pathway regulates the growth and development of organisms via two effector proteins crucial for translation initiation and ribosomal biogenesis by regulating the expression of *eukaryotic translation initiation factor 4E-binding protein* (*4EBP*) and *70 kDa ribosomal protein S6 kinase* (*P70S6K*) [42]. Thus, we further analyzed the expression of *4EBP* and *P70S6K* in the fat body of DHX8-KO silkworm, and the result showed that both *4EBP* and *P70S6K* expressions were significantly down-regulated (Figure 7B). Moreover, the levels of phosphorylated 4EBP and P70S6K were also decreased in the fat body following knockout of *BmDHX8* (Figure 7C). These data suggest that high levels of *BmDHX8* may promote larval growth in silkworm, possibly by activating the mTOR pathway and thereby modulating its downstream effectors, 4EBP and P70S6K.

### 3.8. Knockout of BmDHX8 Causes mRNA Splicing Defects in Target Genes

**Figure 8:**
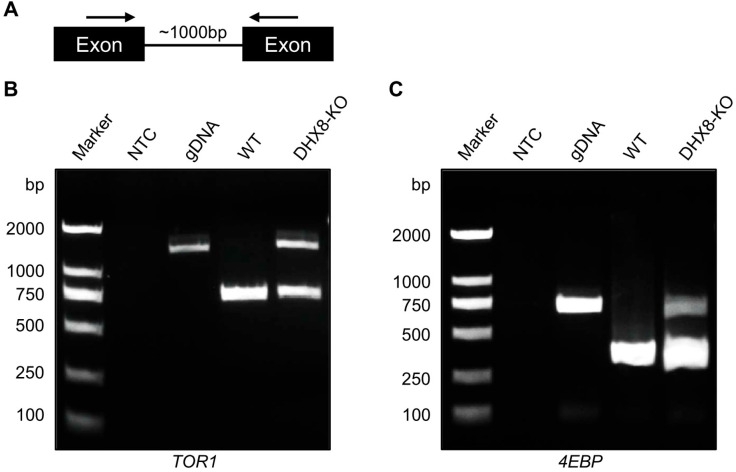
Knockout of *BmDHX8* caused mRNA splicing defects in target genes. (**A**) Schematic diagram of the PCR assay used to assess nuclear splicing. Arrows indicate the locations of the forward and reverse primers. (**B**,**C**) Incomplete splicing of *TOR1* and *4EBP* was detected by RT-PCR in the fat body of DHX8-KO mutants. M: marker, NTC: no template control, gDNA: genomic DNA, WT: cDNA from wild-type individuals, DHX8-KO: cDNA from *BmDHX8* knockout individuals.

DHX8 functions as RNA helicase that is required for the release of mature mRNA from the spliceosome in which mRNA splicing occurs. In yeast and zebrafish, mutation of DHX8 resulted in defective mRNA splicing [16,17]. To investigate whether BmDHX8 is involved in mRNA splicing or not, we performed an RT-PCR assay with primers in adjacent exons from selected genes where introns are less than 1000 bp in length (Figure 8A). Using this criterion, only a limited set of genes from the lipid metabolism and mTOR pathways, including *TOR1* and *4EBP*, were selected for further splicing analysis. As shown in Figure 8B,C, both *TOR1* and *4EBP* genes were not spliced completely in BmDHX8 mutants. Although the set of genes analyzed here is not exhaustive, our findings indicate that BmDHX8 plays a role in spliceosomal mRNA splicing, thereby modulating the abundance of its target transcripts.

## 4. Discussion

The spliceosome, an essential macromolecular complex in eukaryotic cells, catalyzes both constitutive and alternative splicing of intron-containing mRNAs to produce mature transcripts for protein synthesis [5]. As a key component of the spliceosome, DHX8 facilitates the efficient splicing of the pre-mRNA and thereby underpins multiple biological processes [43]. In yeast, Prp22, the functional homologue of DHX8, ensures splicing fidelity via a proofreading mechanism, selectively promoting optimal and rejecting suboptimal splice sites [11,44]. DHX8 also plays a vital role in cell cycle progression. For example, DHX8 knockdown in HeLa cells causes defects in cell division [15], and its targeted knockout in zebrafish leads to severe cell division abnormalities in embryos [16], ultimately compromising cell and embryonic viability. In this study, we investigated the function of DHX8 in silkworms. By using CRISPR-Cas9 to disrupt *BmDHX8* expression, we found that its loss severely impairs silkworm development and leads to mRNA splicing defects.

Our analysis revealed that DHX8 possesses highly conserved amino acid sequences and RNA helicase domains across species, highlighting its fundamental biological importance. Interestingly, subcellular localization assays showed that BmDHX8 forms distinct dot-like structures in the nucleolus, consistent with its role in spliceosome-mediated mRNA processing. Notably, *BmDHX8* expression peaked on day 3 of the fifth instar larval stage, coinciding with a critical developmental period marked by intensive silk protein synthesis and rapid somatic growth [45]. This temporal expression pattern suggests that BmDHX8 may help coordinate nutrient-related gene expression to meet the heightened metabolic demands during this growth phase, as well as during subsequent pupal and adult development. To explore the physiological role of BmDHX8 in silkworms, we generated a BmDHX8 mutant strain. Most mutants displayed significantly reduced body size and developmental delays across larval stage, demonstrating that BmDHX8 is required for normal silkworm development. The observation of dwarfism-like phenotypes in BmDHX8 mutants underscores their potential as a model for investigating human growth disorders, an avenue that merits future investigations.

Insect growth and development depend heavily on lipid storage and utilization, processes centered in the fat body where triglycerides account for over 90% of lipid content [23,24,25]. Our study showed that DHX8-KO mutants exhibited reduced triglyceride levels in the fat body, attributable to the significant down-regulation of key lipid synthesis genes (*FAS*, *LSD1*, *LSD2*, and *LIPIN1*) and up-regulation of lipid catabolism genes (*BMM*, *LIP1*, and *LIP3*). Additionally, BmDHX8 knockout led to dysregulation of upstream hormonal regulators, including the sterol regulatory factor *SREBP* and the adipokinetic hormone receptor *AKHR*, in the fat body, consistent with altered expression of lipid metabolic genes. These findings suggest that SREBP-mediated lipogenesis and AKHR-dependent lipolysis jointly regulate lipid homeostasis in silkworm fat body cells, with BmDHX8 playing a central role in modulating this metabolic balance, a requirement for proper silkworm development.

Beyond lipid metabolism, the mTOR nutrient-sensing pathway regulates organismal nutrition and cell growth in response to amino acids and growth factors [40,41], and also influences lipid metabolism, as illustrated by the lipid-deficient phenotype in *Drosophila* mTOR mutants [46]. Correspondingly, we observed reduced expression of mTOR components (*TOR1* and *TOR2*) in DHX8-KO mutants. Moreover, disruption of *BmDHX8* led to a significant decrease in both transcription and phosphorylation levels of the downstream effectors 4EBP and P70S6K, indicating that BmDHX8 also modulates mTOR signaling. Previous studies have shown that mTOR signaling inhibition down-regulates *SREBP* [47], consistent with our observation of reduced *SREBP* expression in BmDHX8 knockout silkworms. This points to a complex regulatory interplay among DHX8, mTOR signaling, and SREBP in controlling downstream gene expression. While this proposed regulatory axis represents an integrative hypothesis derived from our transcript and phosphorylation data, further functional studies are needed to validate these interactions and establish causality. Overall, our work suggests that disruption of *BmDHX8* impairs protein synthesis and lipid accumulation, leading to reduced body and tissue size in silkworms. This reduction in tissue size in DHX8-KO mutants likely further limits dietary nutrient absorption, thereby exacerbating developmental impairment.

BmDHX8 shares sequence homology with human DHX8 and yeast Prp22, an essential ATP-dependent helicase involved in mRNA metabolism [11,17,44]. In yeast, Prp22 promotes the second splicing step by facilitating branch point-exon ligation (for introns ≥21 bp) and catalyzing transesterification, while also ensuring splicing fidelity and mediating mRNA release from the spliceosome [13,17]. Given this evolutionarily conserved role, our study demonstrates that knockout of *BmDHX8* impairs the splicing of key regulatory genes, particularly components of the mTOR signaling pathway including *TOR1* and *4EBP*, highlighting its critical function in RNA processing in silkworms. Notably, this disruption appears to be specific to certain transcripts rather than reflecting a global splicing defect across the transcriptome. Future research should aim to elucidate the molecular mechanism by which BmDHX8 recognizes and processes its target pre-mRNAs, thereby bridging its spliceosomal function with systemic metabolic regulation.

Taken together, our findings identify BmDHX8 as a key developmental regulator in silkworms, linking spliceosomal function to nutrient homeostasis. Disruption of *BmDHX8* leads to pre-mRNA splicing defects that underlie observed metabolic impairments, including altered lipid homeostasis and nutrient-responsive signaling. In addition to confirming the evolutionarily conserved helicase activity of DHX8, this study uncovers its specific role in the post-transcriptional regulation of nutrient metabolic pathways, providing novel insights into nutrient-response systems in Lepidoptera.

## 5. Conclusions

Our study demonstrates that BmDHX8 plays a critical role in silkworm larval development through its conserved function in pre-mRNA splicing. The observed splicing defects and associated developmental impairments highlight the importance of DHX8 in integrating RNA processing with metabolic regulation, indicating that BmDHX8 mutant silkworms could serve as a useful model for investigating growth defects and metabolic disorders. Although these findings have broader implications for understanding growth and metabolic control, the present study is confined to lepidopteran silkworms. Therefore, future comparative studies across other species are needed to definitively establish the generality of these mechanisms.

## Data Availability

The original contributions presented in the study are included in the article, further inquiries can be directed to the corresponding author.

## Supplementary Materials

The following supporting information can be downloaded at https://www.mdpi.com/article/10.3390/insects17030236/s1, Figure S1: Amplification of the full-length CDS of BmDHX8 in silkworm; Figure S2: Protein structures in DHX8-KO mutations related to Figure 3D; Table S1: List of primers used in this study.
