# Supplementary material for: DHX8 Plays a Critical Role in Larval Development in Lepidopteran Bombyx mori
Source: Insects. 2026 Feb 25;17(3):236. doi: 10.3390/insects17030236 (PMC13027099; doi:10.3390/insects17030236)
Supplement: Supplementary file 1 [file insects-17-00236-s001.zip › Table S1.pdf]

Supporting Information

Table S1. List of primers used in this study.

Gene name	Sequence (5'-3')
DHX8-gRNA-F	AAGTGATGGTACAATGTTTGCAACG
DHX8-gRNA-R	AAACCGTTGCAAACATTGTACCATC
DHX8-ORF-F	TTCTCTAAGGAAATACTTAACCATGGACGAAGTCACTAAATTAGAGC
DHX8-ORF-R	GCTGGGTCTAGATATCTCGAGCTAATTTCTTCTGCGTCTGACTCT
DHX8-qPCR-F	GTTTATATACATCCTTCCAGTGCACTGTTC
DHX8-qPCR-R	AAACTCAACCAGCCATTTCGGGTCTATGGT
FAS-qPCR-F	GATCAGAGCTGGCCAAGCTGAGC
FAS-qPCR-R	CTTCAGCACCGCCTGCAGCAGC
LSD1-qPCR-F	CGTTCGAATACACCACGAAAAT
LSD1-qPCR-R	ATCCTTGACTCTGACTAACAGC
LSD2-qPCR-F	GATCAACGAACTCAGAAACTCG
LSD2-qPCR-R	TTAGAAGGATCCACGGATTTCG
BMM-qPCR-F	GGATGGAGGGTTCAGTGATAAC
BMM-qPCR-R	TGTTCGCTAGGTTTACGTGG
LIP1-qPCR-F	CCAAGATTTGGAAACCAGAA
LIP1-qPCR-R	GTGACGATCAGGTCCTTGGT
LIP3-qPCR-F	GCCACGACGAAAAACAATTT
LIP3-qPCR-R	AGTTGCCTTCTGCTCCGTAA
LIPIN1-qPCR-F	TTTGTGCATATCAAGCTGTCGG
LIPIN1-qPCR-R	CCAGCACTGACATATGGGAATA
SREBP-qPCR-F	TCTGAGGAGGCTGTCACAGAGC
SREBP-qPCR-R	CCGCATATTATAGACGCTCGGTT
AKHR-qPCR-F	CACGAGAACCTTCGGACTT
AKHR-qPCR-R	CACGCATTGATTGTAACCT
TOR1-qPCR-F	TAAAAAGACGAACAAAACGGCG
TOR1-qPCR-R	GAAAACCTCCACCTTATGCTTC
TOR2-qPCR-F	GCTCAGTTACTTGCCGAATTAG
TOR2-qPCR-R	GATGCGAACTTAAGCCAAGTAG
P70S6K-qPCR-F	TGCCAACTTAATGTTCCAGGGA
P70S6K-qPCR-R	ATGAGGTCCTCTTGTTGAGCGT

4EBP-qPCR-F	ATGTCAGCGTCACCTATC
4EBP-qPCR-R	CACTATTCTTGTACCTCCAG
eIF-4a-qPCR-F	TTCGTACTGGCTCTTCTCGT
eIF-4a-qPCR-R	CAAAGTTGATAGCAATTCCCT
TOR1-RT-F	TGAAAATTTCTGACAAAGGCGAC
TOR1-RT-R	ATCCGTTACGTTTTTTCTGTGTT
4EBP-RT-F	TGTCAGCGTCACCTATCGCG
4EBP-RT-R	TTAGAGATCCATGCTGAAGGTT
