# Supplementary figures and images for: DHX8 Plays a Critical Role in Larval Development in Lepidopteran Bombyx mori
Source: Insects. 2026 Feb 25;17(3):236. doi: 10.3390/insects17030236 (PMC13027099; doi:10.3390/insects17030236)

Supporting Information  
Figure S1

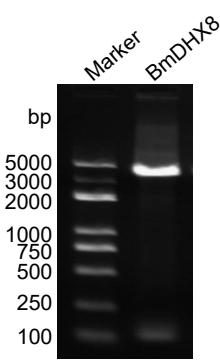

Supporting Information  
Figure S2

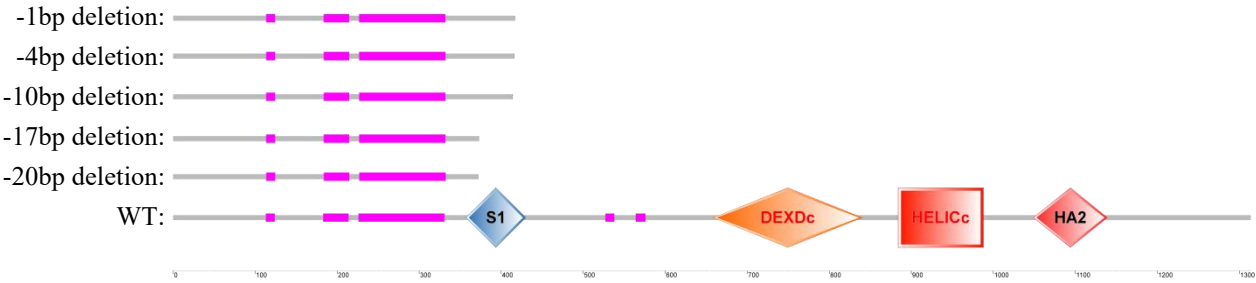

Supplement: Supplementary file 1 [file insects-17-00236-s001.zip › Figure S1.pdf]
